# A Virtual Reality Soldier Simulator with Body Area Networks for Team Training

**DOI:** 10.3390/s19030451

**Published:** 2019-01-22

**Authors:** Yun-Chieh Fan, Chih-Yu Wen

**Affiliations:** 1 Simulator Systems Section, Aeronautical System Research Division, National Chung-Shan Institute of Science and Technology, Taichung 407, Taiwan; d105064101@mail.nchu.edu.tw; 2 Department of Electrical Engineering, Innovation and Development Center of Sustainable Agriculture (IDCSA), National Chung Hsing University, Taichung 402, Taiwan

**Keywords:** virtual reality, body area network, training simulator

## Abstract

Soldier-based simulators have been attracting increased attention recently, with the aim of making complex military tactics more effective, such that soldiers are able to respond rapidly and logically to battlespace situations and the commander’s decisions in the battlefield. Moreover, body area networks (BANs) can be applied to collect the training data in order to provide greater access to soldiers’ physical actions or postures as they occur in real routine training. Therefore, due to the limited physical space of training facilities, an efficient soldier-based training strategy is proposed that integrates a virtual reality (VR) simulation system with a BAN, which can capture body movements such as walking, running, shooting, and crouching in a virtual environment. The performance evaluation shows that the proposed VR simulation system is able to provide complete and substantial information throughout the training process, including detection, estimation, and monitoring capabilities.

## 1. Introduction

In recent years, since virtual reality (VR) training simulators allow soldiers to be trained with no risk of exposure to real situations, the development of a cost-effective virtual training environment is critical for training infantry squads [1,2,3]. This is because if soldiers do not develop and sustain tactical proficiency, they will not be able to react in a quickly evolving battlefield. To create a VR military simulator that integrates immersion, interaction, and imagination, the issue of how to use VR factors (e.g., VR engine, software and database, input/output devices, and users and tasks) is an important one. The VR simulator is able to integrate the terrain of any real place into the training model, and the virtual simulation trains soldiers to engage targets while working as a team [4,5].

With a head-mounted display (HMD), the key feature used in VR technology, soldiers are immersed in a complex task environment that cannot be replicated in any training areas. Visual telescopes are positioned in front of the eyes, and the movement of the head is tracked by micro electro mechanical system (MEMS) inertial sensors. Gearing up with HMDs and an omnidirectional treadmill (ODT), soldiers can perform locomotive motions without risk of injury [6,7,8]. Note that the above systems do not offer any posture or gesture interactions between the soldiers and the virtual environment. To address this problem, the authors of [9,10,11,12,13,14,15,16,17] proposed an action recognition method based on multiple cameras. To tackle the occlusion problem, the authors mostly used Microsoft Kinect to capture color and depth data from the human body, which requires multiple devices arranged in a specific pattern for the recognizing different human actions. Therefore, the above methods are not feasible in a real-time virtual environment for military training.

To overcome the limitations of the real-time training system, the dismounted soldier training system (DSTS) was developed [18,19,20,21]. In the DSTS, soldiers wear special training suits and HMDs, and stand on a rubber pad to create a virtual environment. However, the inertial sensors of training suits can only recognize simple human actions (e.g., standing and crouching). When soldiers would like to walk or run in the virtual environment, they have to control small joysticks and function keys on simulated rifles, which are not immersive for locomotion. Thus, in a training situation, soldiers will not be able to react quickly when suddenly encountering enemy fire. As a result, though the DSTS provides a multi-soldier training environment, it has weaknesses with respect to human action recognition. Therefore, in order to develop a training simulator that is capable of accurately capturing soldiers’ postures and movements and is less expensive and more effective for team training, body area networks (BANs) represent an appropriate bridge for connecting physical and virtual environments [22,23,24,25,26], which contain inertial sensors like accelerometers, e-compasses and gyroscopes to capture human movement. 

Xsens Co. have been developing inertial BANs to track human movement for several years. Although the inertial BAN, called the MVN system, is able to capture human body motions wirelessly without using optical cameras, the 60 Hz system update rate is too slow to record fast-moving human motion. The latest Xsens MVN suit solved this problem, reaching a system update rate of 240 Hz. However, the MVN suit is relatively expensive in consumer markets. In addition, it is difficult to support more than four soldiers in a room with the MVN system, since the wireless sensor nodes may have unstable connections and high latency. On the other hand, since the Xsens MVN system is not an open-source system, the crucial algorithm of the inertial sensors does not adaptively allow the acquisition by and integration with the proposed real-time program. Moreover, the MVN system is only a motion-capture BAN and is not a fully functional training simulator with inertial BANs [26]. 

**Figure 1:**
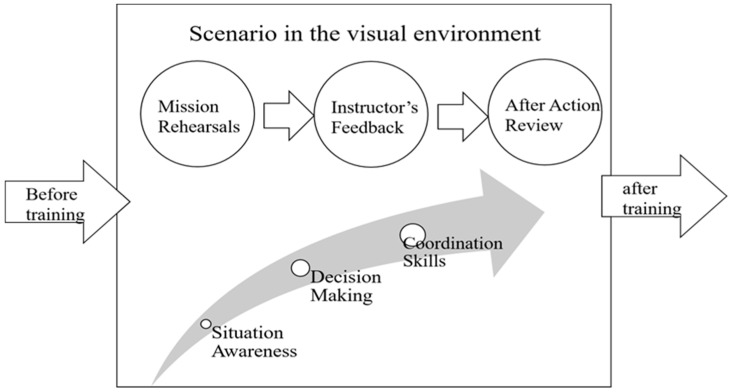
System training effectiveness process.

For the above-mentioned reasons, this paper proposes a multi-player training simulator that immerses infantry soldiers in the battlefield. The paper presents a technological solution for training infantry soldiers. Fully immersive simulation training provides an effective way of imitating real situations that are generally dangerous, avoiding putting trainees at risk. To achieve this, the paper proposes an immersive system that can immediately identify human actions. Training effectiveness in this simulator is highly remarkable in several respects:

1. *Cost-Effective Design*: We designed and implemented our own sensor nodes, such that the fundamental operations of the inertial sensors could be adaptively adjusted for acquisition and integration. Therefore, it has a competitive advantage in terms of system cost.
2. *System Capacity*: Based on the proposed simulator, a six-man squad is able to conduct military exercises that are very similar to real missions. Soldiers hold mission rehearsals in the virtual environment such that leaders can conduct tactical operations, communicate with their team members, and coordinate with the chain of command.
3. *Error Analysis*: This work provides an analysis of the quaternion error and further explores the sensing measurement errors. Based on the quaternion-driven rotation, the measurement relation with the quaternion error between the earth frame and the sensor frame can be fully described.
4. *System Delay Time:* The update rate of inertial sensors is about 160 Hz (i.e., the refresh time of inertial sensors has a time delay of about 6 ms). The simulator is capable of training six men at a 60 Hz system update rate (i.e., the refresh time of the entire system needs about 16 ms), which is acceptable for human awareness with delay (≤40 ms).
5. *System Feedback*: Instructors can provide feedback after mission rehearsals using the visual after action review (AAR) function in the simulator, which provides different views of portions of the action and a complete digital playback of the scenario, allowing a squad to review details of the action. Furthermore, instructors can analyze data from digital records and make improvements to overcome the shortcomings in the action (Figure 1). Accordingly, in the immersive virtual environment, soldiers and leaders improve themselves with respect to situational awareness, decision-making, and coordination skills.

## 2. System Description

**Figure 2:**
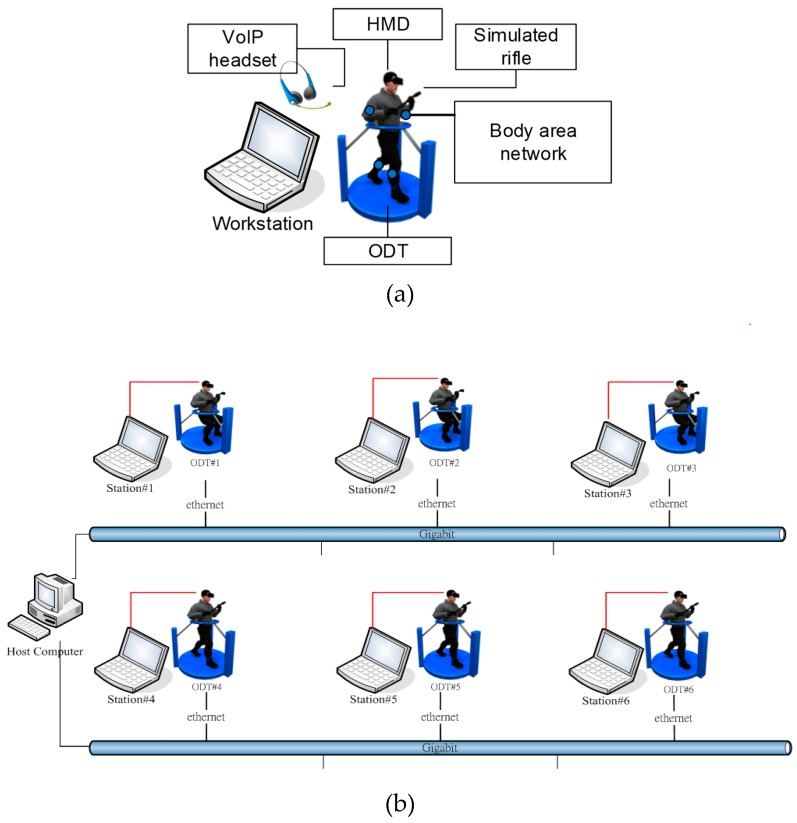
System architecture: single-soldier layout (**a**); multi-soldier network (**b**).

**Figure 3:**
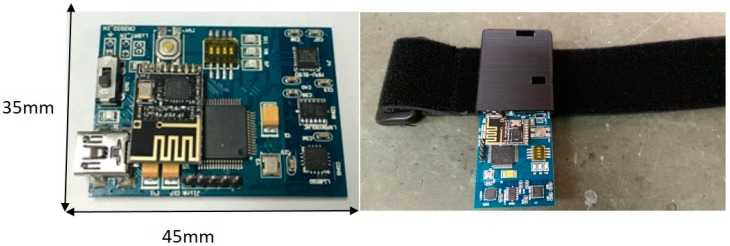
Top view of sensor nodes (**Left**); the wearable sensor node (**Right**).

**Figure 4:**
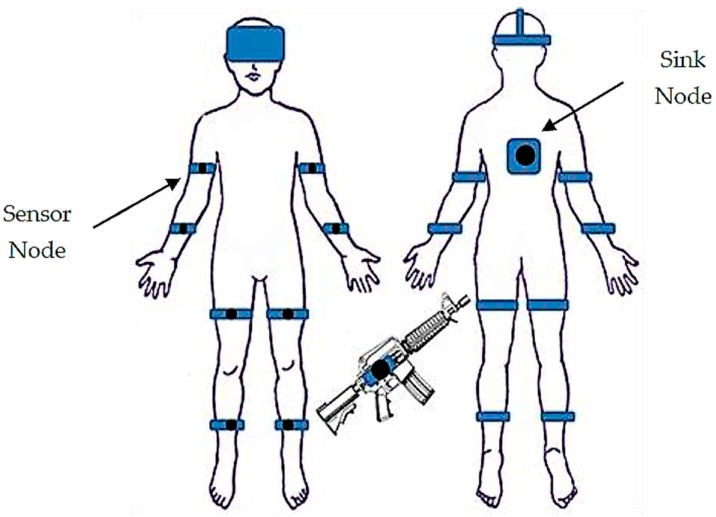
Deployment of sensor nodes and the sink node.

The proposed system, which is deployed in five-square-meter area (Figure 2), is a robust training solution that can support up to six soldiers. The virtual reality environment is implemented in C++ and DirectX 11. In addition to the virtual system, each individual soldier stands in the center of an ODT. The ODTs are customized hardware designed for the soldier training simulator, which is equipped with a high-performance workstation that generates the visual graphics for the HMD and provides voice communication functions based on VoIP technology. The dimensions of the ODT are 47” x47 “x78”, 187 lbs. The computing workstation on the ODT is equipped with a 3.1 GHz Intel Core i7 processor, 8 GB RAM, and an NVidia GTX 980M graphics card. The HMD is an Oculus Rift DK2, which has two displays with 960 × 1080 resolution per eye. The VoIP software is implemented based on Asterisk open-source PBX. Soldiers are not only outfitted with HMDs, but also equipped with multiple inertial sensors, which consist of wearable sensor nodes (Figure 3) deployed over the full body (Figure 4). As depicted in Figure 3, a sensor node consists of an ARM-based microcontroller and nine axial inertial measurement units (IMUs), which are equipped with ARM Cotex-M4 microprocessors and ST-MEMS chips. Note that these tiny wireless sensor nodes can work as a group to form a wireless BAN, which uses an ultra-low power radio frequency band of 2.4G. 

### 2.1. Sensor Modeling

This subsection the sensors are modeled in order to formulate the orientation estimation problem. The measurement models for the gyroscope, accelerometer, and magnetometer are briefly discussed in the following subsections [27].

**Figure 5:**
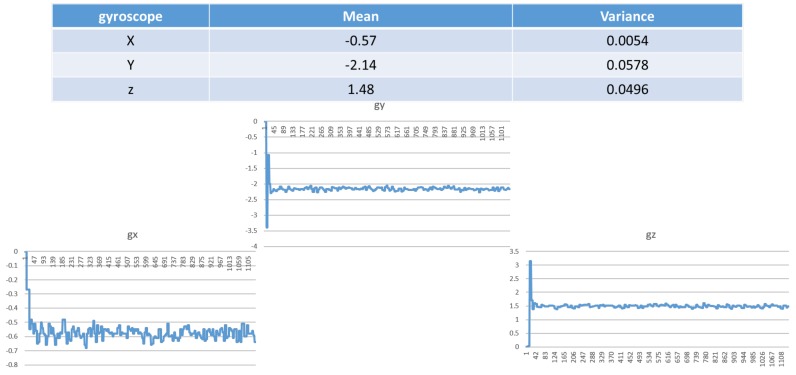
Mean and variance of the gyroscope in the x, y and z directions.

*(1) Gyroscope:* The measured gyroscope signal ${}^{s}\omega_{t}^{m}\;$ can be represented in the sensor frame *s* at time *t* using

(1) $${}^{s}\omega_{t}^{m}={}^{s}\omega_{t}+b_{\omega ,\;t}+e_{\omega ,\;t}^{}$$

where ${}^{s}\omega_{t}$ is the true angular velocity, $b_{\omega ,\;t}$ is the bias caused by low-frequency offset fluctuations, and the measurement error $e_{\omega ,\;t}$ is assumed to be zero-mean white noise. As shown in Figure 5, the raw data of the measured gyroscope signals in the x, y and z directions includes true angular velocity, bias and error. 

**Figure 6:**
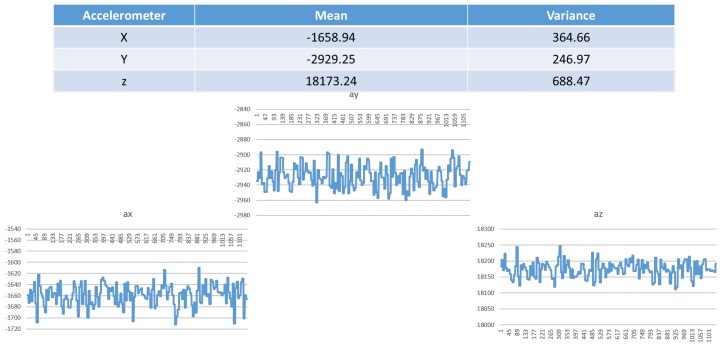
Mean and variance of the accelerometer in the x, y and z directions.

*(2) Accelerometer:* Similar to the gyroscope signal, the measured accelerometer signal ${}^{s}a_{t}^{m}$ can be represented using

(2) $${}^{s}a_{t}^{m}\approx {}^{s}a_{b,t}+b_{a,\;t}+e_{a,\;t}$$

where ${}^{s}a_{b,\;t}$ is the linear acceleration of the body after gravity compensation, $b_{a,\;t}$ is the bias caused by low-frequency offset fluctuations, and the measurement error $e_{a,\;t}$ is assumed to be zero-mean white noise. As shown in Figure 6, the raw data of the measured accelerometer signals in the x, y and z directions includes true linear acceleration, offset and error.

*(3) Magnetometer:* For the magnetometer, the measured signal ${}^{s}m_{t}^{m}$ is often modeled as the sum of the earth’s magnetic field ${}^{s}m_{t},\;\mathrm{magnetic}\;\mathrm{offset}\;b_{m,\;t},$ and noise $e_{m,\;t}$, which yields

(3) $${}^{s}m_{t}^{m}\approx {}^{s}m_{t}+b_{m,\;t}+e_{m,\;t}$$

**Figure 7:**
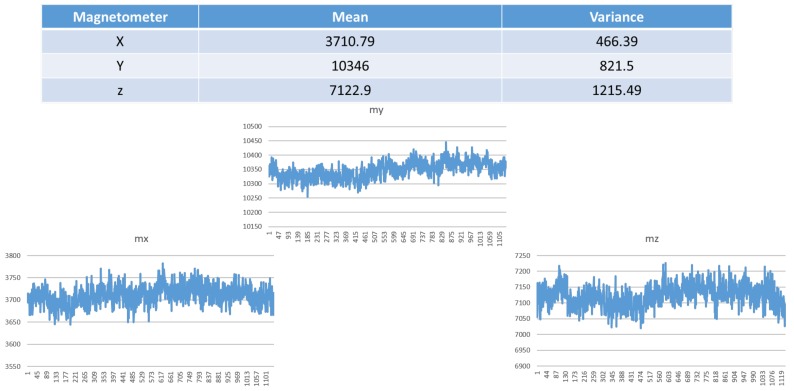
Mean and variance of the magnetometer in the x, y and z directions.

As shown in Figure 7, the raw data of the measured magnetometer signals in the x, y and z directions includes the earth’s magnetic field, offset and error.

### 2.2. Sensor Calibration

Since the accuracy of the orientation estimate depends heavily on the measurements, the null point, and the scale factor of each axis of the sensor, a calibration procedure should be performed before each practical use. To this end, the sensor calibration is described as follows:

- Step 1: Given a fixed gesture, we measure the sensing data (i.e., the raw data) and calculate the measurement offsets.
- Step 2: Remove the offset and normalize the modified raw data to the maximum resolution of the sensor’s analog-to-digital converter. In this work, the calibrated results (CR) of the sensors are described by Equations (4)–(6).

Table 1 shows the means of the calibrated results in the x, y and z directions of sensor nodes, which include the accelerometer, the magnetometer, and the gyroscope. Note that the inertial sensor signals (e.g., ${}^{s}a_{t}^{m}$, ${}^{s}m_{t}^{m}$, and ${}^{s}\omega_{t}^{m}$) are measured without movement, and that a small bias or offset (e.g., $b_{a,\;t}$ or $b_{m,\;t}$ or $b_{\omega ,\;t}$) in the measured signals can therefore be observed. In general, the additive offset that needs to be corrected for before calculating the estimated orientation is very small. For instance, the additive offset would be positive or negative in the signal output (e.g., the average $b_{\omega ,\;t}=-0.08$ degree/sec and ${}^{s}\omega_{t}^{m}=0$ degree/sec) without movement.

(4) $${\mathrm{CR}}_{acc}=\left({}^{s}a_{t}^{m}\pm b_{a,\;t}\right)/\max\left({}^{s}a_{t}^{m}\right)$$

(5) $${\mathrm{CR}}_{mag}=\left({}^{s}m_{t}^{m}\pm b_{m,\;t}\right)/\max\left({}^{s}m_{t}^{m}\right)$$

(6) $${\mathrm{CR}}_{gyro}=\left({}^{s}\omega_{t}^{m}\pm b_{\omega ,\;t}\right)/\max\left({}^{s}\omega_{t}^{m}\right)$$

After bias error compensation for each sensor, the outputs of the sensors represented in (1)–(3) can be rewritten as ${}^{s}\omega_{t}^{m}={}^{s}\omega_{t}+e_{\omega ,\;t}$, ${}^{s}a_{t}^{m}\approx {}^{s}a_{b,t}+e_{a,\;t}$, and ${}^{s}m_{t}^{m}\approx {}^{s}m_{t}+e_{m,\;t}$, and they are then applied as inputs for the simulation system. In particular, the proposed algorithm can be good at handling zero-mean white noise. After the completion of modeling and calibration of the sensor measurements from the newly developed MARG, all measurements are then represented in the form of quaternions as inputs of the proposed algorithm. 

**Figure 8:**
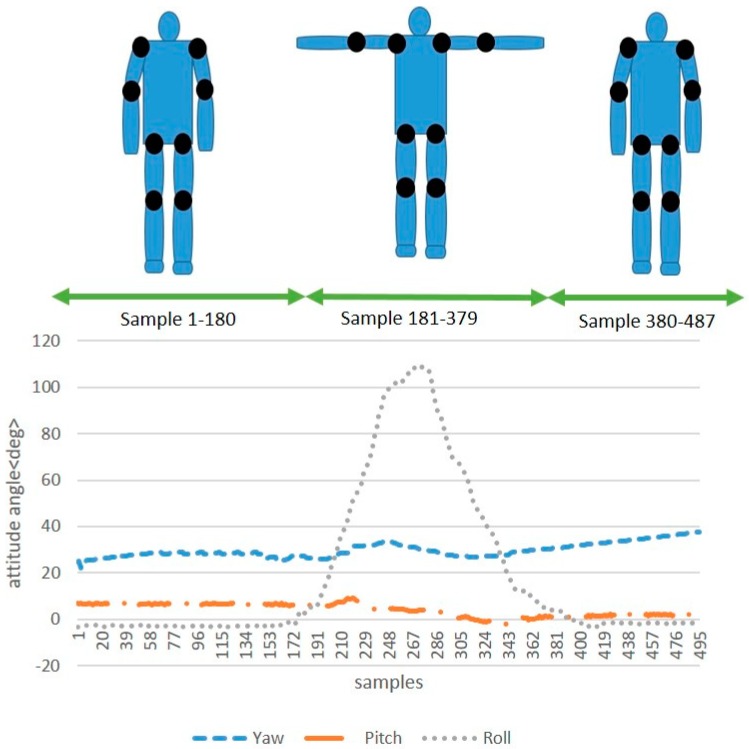
The attitude angles of a sensor node placed on the upper arm when standing in a T-pose.

As shown in Figure 8, a typical run of the attitude angles of a sensor node placed on the upper arm when standing in a T-pose is recorded. In the first step, we stand with the arms naturally at the sides. Then, we stretch the arms horizontally with thumbs forward, and then we move back to the first step. This experiment shows that the sensor node is capable of tracking the motion curve directly.

### 2.3. Information Processing

**Figure 9:**
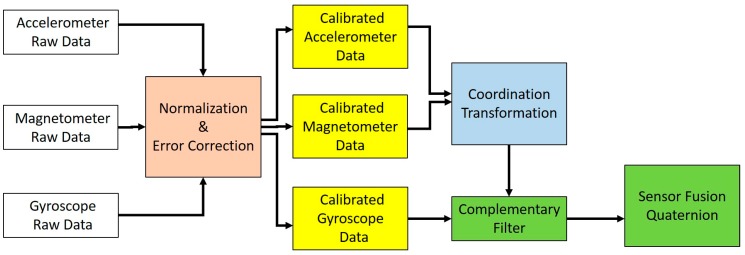
The diagram shows the process of sensor fusion.

As mentioned above, human action recognition is crucial to developing an immersive training simulator. The wireless BAN is able to instantaneously track the action of the skeleton of a soldier. The microcontroller of the sensor node acquires the raw data from the accelerometer, gyroscope and magnetometer through SPI and I2C interfaces. The measurements of the accelerometer, gyroscope and magnetometer are contaminated by errors of scale factor and bias, resulting in a large estimation error during sensor fusion. Thus, a procedure for normalization and error correction is needed for the sensor nodes to ensure the consistency of the nine-axis data (Figure 9). After that, the calibrated accelerometer and magnetometer data can be used to calculate the orientation and rotation. Estimation based on the accelerometer does not include the heading angle, which is perpendicular to the direction of gravity. Thus, the magnetometer is used to measure the heading angle. the Euler angles are defined as follows:

Roll−Φ: rotation about the X-axisPitch−θ: rotation about the Y-axisHeading−ψ: rotation about the Z-axis.

Let *X_acc_*, *Y_acc_*, *Z_acc_* be the accelerometer components. We have:

(7) $$\mathrm{Pitch}\;\theta =\tan^{-1}\left(\frac{-X_{acc}}{\sqrt{Z_{acc}{}^{2}+Y_{acc}{}^{2}}}\right)$$

(8) $$\mathrm{Roll}\;\Phi =\tan^{-1}\left(\frac{Y_{acc}}{Zacc}\right)$$

Let *X_mag_*, *Y_mag_*, *Z_mag_* be the magnetometer components. We have:

(9) $$X_{h}=X_{mag}\cos\left(\theta \right)+Y_{mag}\sin\left(\theta \right)\times \sin\left(\Phi \right)+Z_{mag}\sin\left(\theta \right)\times \cos\left(\Phi \right)$$

(10) $$Y_{h}=-Y_{mag}\cos\left(\Phi \right)+Z_{mag}\sin\left(\Phi \right)$$

(11) $$\mathrm{Heading}\;\psi =\tan^{-1}\left(\frac{Y_{h}}{X_{h}}\right)$$

Accordingly, the complementary filter output (using gyroscope) is

(12) $$Angle_{output}=W\times (Angle_{output}+Angle_{gyro})+\left(1-W\right)\times Angle_{acc+mag},\;\;0<W<1$$

To mitigate the accumulated drift from directly integrating the linear angular velocity of the gyroscope, the accelerometer and magnetometer are used as aiding sensors to provide the vertical and horizontal references for the Earth. Moreover, a complementary filter [28,29] is able to combine the measurement information of an accelerometer, a gyroscope and a magnetometer, offering big advantages in terms of both long-term and short-term observation. For instance, an accelerometer does not drift over the course of a long-term observation, and a gyroscope is not susceptible to small forces during a short-term observation. The W value of the complementary filter in Equation (12) is the ratio in which inertial sensor data is fused, which can then be used to compensate for gyroscope drift by using the accelerometer and magnetometer measurements. We always set the W value to 0.9, or probably even higher than 0.9. Therefore, the complementary filter provides an accurate orientation estimation. 

### 2.4. Communication and Node Authentication Procedures

To tackle the problem of interference and to reduce the bit error rate (BER) of the wireless data transmission between the sensor nodes and the sink node, communication mechanisms can be applied to build a robust wireless communication system [30,31]. The communication technique employed for communication between the sensor nodes and the sink node is frequency hopping spread spectrum (FHSS). The standard FHSS modulation technique is able to avoid interference between different sensor nodes in the world-wide ISM frequency band, because there are six BANs in total, all of which are performing orientation updates in a small room. Moreover, the sensor nodes’ transmission of data to the sink node is based on packet communication. 

**Figure 10:**
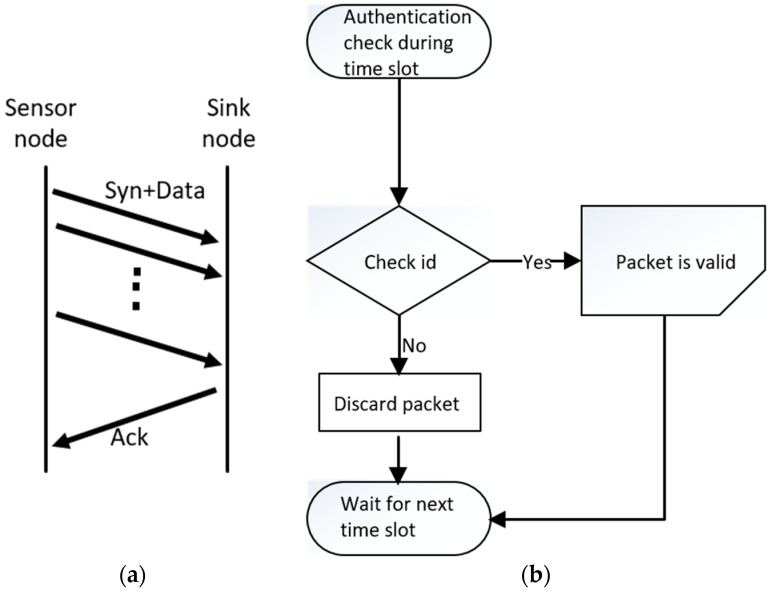
Operation mode during each time slot. (**a**) Step 1: automatic packet synchronization; (**b**) Step 2: identification check on valid packets by the sink node.

**Figure 11:**
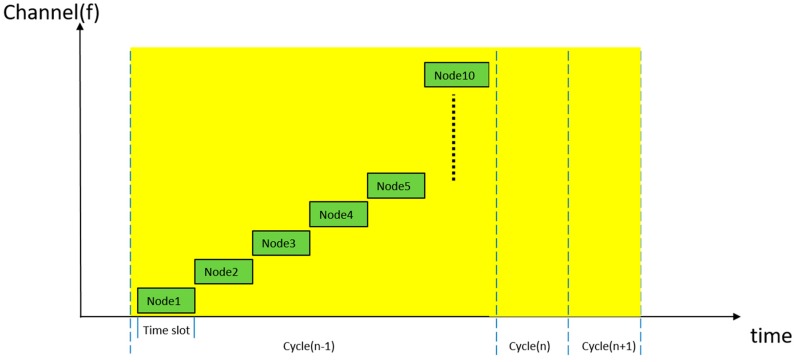
The diagram shows that sensor nodes communicate with sink node in two domains.

In the beginning, sensor nodes send packets, which include orientation and rotation data. When the sink node receives a packet from a sensor node, an acknowledgement packet will be transmitted immediately (Figure 10). If the acknowledgement fails to arrive, the sensor node will retransmit the packet, unless the number of retries exceeds the retransmission limit. When the sink node receives packets in operation mode, it will check the payload of the packets. As shown in Figure 11, if the payload is equal to the sensor node’s unique identification information, the sink node will accept the packet and scan the next sensor node’s channel during the next time slot. The ten wireless sensor node channels operate at a frequency range of 2400–2525 GHz.

### 2.5. System Intialization

**Figure 12:**
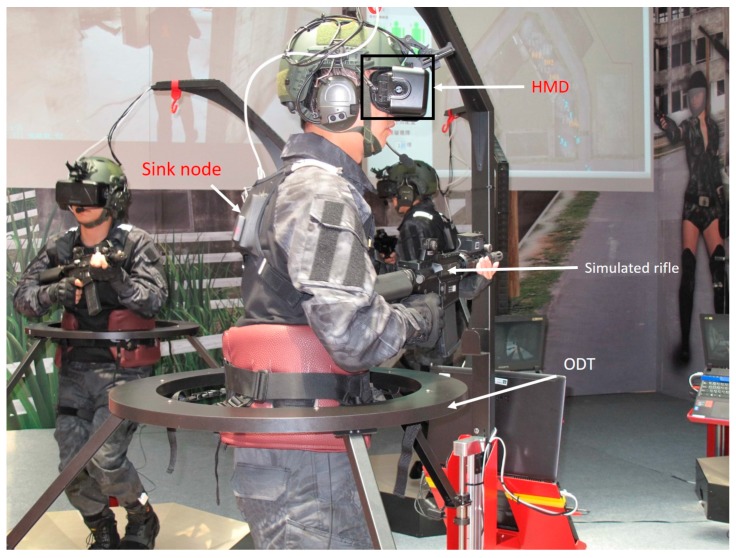
A fully equipped soldier.

The sink node is deployed on the back of the body and collects the streaming data from multiple wireless sensor nodes. Afterwards, the streaming data from the sensor nodes is transmitted to the workstation via Ethernet cables, modeling the skeleton of the human body along with the structure of the firearm (Figure 12). Table 2 describes the data structure of a packet, which is sent by a sensor node to the sink node in every ODT. Table 3 shows the integrated data that describes the unique features of each soldier in the virtual environment. The description provides the appearance characteristics, locomotion, and behaviors of each soldier [32,33].

For sensor nodes, the data stream reported to the sink node is interpreted as the movement-based data structures of a skeleton. Each wearable sensor node of the skeleton has a unique identification information during the initialization phase. As a result, the sink node can distinguish which sensor nodes are online. Accordingly, when turning the power on, the HMDs on the soldiers are automatically connected to the workstations, and the voice communication group setting is immediately ready. Please note that the T-pose calibration is performed in sensor node *n* of a skeleton for initializing the root frame, which is given by

(13) $$q_{1,n}=q_{T,n}⊗q_{0,n}⊗q_{T,n}^{⋆},$$

where $q_{T,n}$ is the reading of sensor node *n* in the modified T-pose and $q_{1,n}$ is the new body frame from the initial root frame $q_{0,n}$.

**Figure 13:**
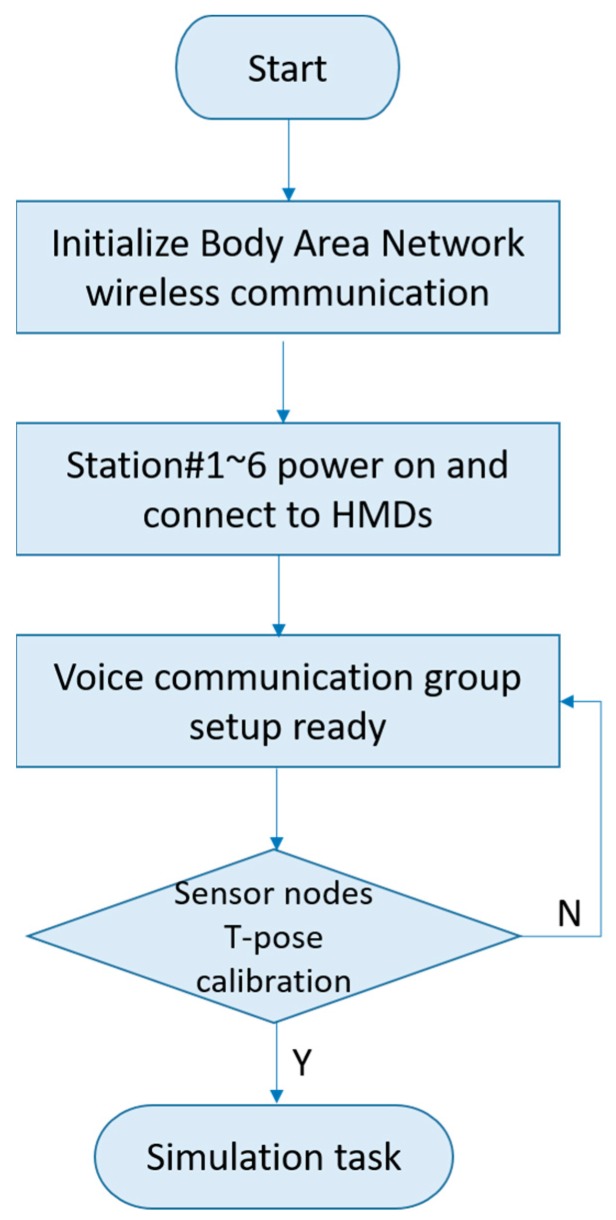
System initialization flow diagram.

Although sensor nodes are always worn on certain positions of a human body, the positions of sensor nodes may drift due to the movements occurring during the training process. Hence, the T-pose calibration procedure can be applied to estimate the orientation of the sensors. After that, the system is prepared to log in for simulation tasks. The system initialization flow diagram is shown in Figure 13.

## 3. Quaternion Representation

This section outlines a quaternion representation of the orientation of the sensor arrays. Quaternions provide a convenient mathematical notation for representing the orientation and rotation of 3D objects because quaternion representation is more numerically stable and efficient compared to rotation matrix and Euler angel representation. According to [34,35,36], a quaternion can be thought of as a vector with four components,

(14) $$q=q_{0}+q_{x}i+q_{y}j+q_{z}k$$

as a composite of a scalar and ordinary vector. The quaternion units $q_{x}$, $q_{y}$, $q_{z}$ are called the vector part of the quaternion, while $q_{0}$ is the scalar part. The quaternion can frequently be written as an ordered set of four real quantities,

(15) $$q=\left[q_{0},\;q_{x},\;q_{y},\;q_{z}\right].$$

Denote ${}_{s}^{e}q$ as the orientation of the earth frame ***u_e_*** with respect to the sensor frame ***u_s_***. The conjugate of the quaternion can be used to represent an orientation by swapping the relative frame, and the sign * denotes the conjugate. Therefore, the conjugate of ${}_{s}^{e}q$ can be denoted as

(16) $${}_{s}^{e}q{}^{*}={}_{e}^{s}q=\left[q_{0},\;-q_{x},\;-q_{y},\;-q_{z}\right].$$

Moreover, the quaternion product ⊗ can be used to describe compounded orientations, and their definition is based on the Hamilton rule in [37]. For example, the compounded orientation ${}_{h}^{s}q$ can be defined by

(17) $${}_{h}^{s}q={}_{e}^{s}q⊗{}_{h}^{e}q,$$

where ${}_{h}^{e}q$ denotes the orientation of the earth frame ***u_e_*** with respect to the frame ***u_h_***.

A human body model consists of a set of body segments connected by joints. For upper limbs and lower limbs, kinematic chains are modeled that branch out around the torso. The kinematic chain describes the relationship between rigid body movements and the motions of joints. A forward kinematics technique, which was introduced for the purposes of robotic control, is used to configure each pair of adjacent segments. In the system, the aim of building human kinematic chains is to determine the transformation matrix of a human body from the first to the last segments and to find the independent configuration for each joint and the relationship with the root frame. 

Thus, the rotation matrix $q_{n-1}^{n}$ used for orientation from sensor node *n*-1 to sensor node *n* is given by

(18) $$q_{n-1}^{n}=q_{n-1}⊗q_{n}^{⋆}.$$

**Figure 14:**
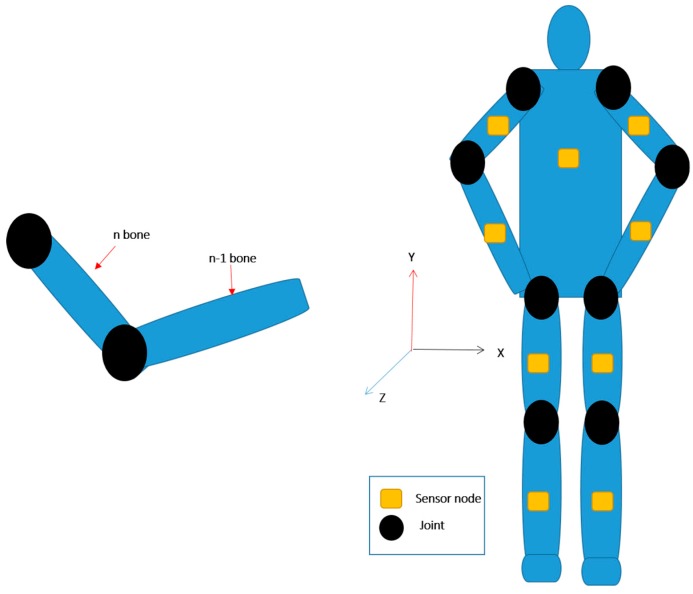
The kinematic chain of a human body.

Figure 14 shows the simplified segment biomechanical model of the human body. The kinematics of segments on which no inertial sensors are attached (e.g., hand, feet, toes) are considered to be rigid connections between neighboring segments. The transformation matrix is defined as

(19) $$Q_{n-1}^{n}=\left[\begin{array}{cc} q_{n-1}^{n} & T_{n-1}^{n}\\ 0 & 1 \end{array}\right].$$

where $T_{n-1}^{n}$ is the translation matrix from sensor frame to body frame. According to [37,38], therefore, the transformation matrix of a human body from the first segment to the n-th segment is

(20) $$Q_{1}^{n}=Q_{n-1}^{n}Q_{n-2}^{n-1}Q_{n-3}^{n-2}\cdots Q_{2}^{3}Q_{1}^{2}.$$

## 4. Performance Analysis

The analysis focuses on the quaternion error and further explores the sensing measurement errors. Based on the quaternion-driven rotation, the measurement relation with the quaternion error between the earth frame and sensor frame can be further described.

### 4.1. Rotation Matrix

According to [36], given a unit quaternion ***q*** = $q_{r}$ + $q_{x}$*i* + $q_{y}$*j* + $q_{z}$*k*, the quaternion-driven rotation can be further described by the rotation matrix ***R***, which yields

(21) $$R=\left[\begin{array}{ccc} 1-2\left(q_{y}{}^{2}+q_{z}{}^{2}\right) & 2q_{x}q_{y}-2q_{r}q_{z} & 2q_{x}q_{z}+2q_{r}q_{y}\\ 2q_{x}q_{y}+2q_{r}q_{z} & 1-2\left(q_{x}{}^{2}+q_{z}{}^{2}\right) & 2q_{y}q_{z}-2q_{r}q_{x}\\ 2q_{x}q_{z}-2q_{r}q_{y} & 2q_{y}q_{z}+2q_{r}q_{x} & 1-2\left(q_{x}{}^{2}+q_{y}{}^{2}\right) \end{array}\right].$$

Let $\hat{q}$ be an estimate of the true attitude quaternion q. The small rotation from the estimated attitude, $\hat{q}$, to the true attitude is defined as $q_{err}$. The error quaternion is small but non-zero, due to errors in the various sensors. The relationship is expressed in terms of quaternion multiplication as follows:

(22) $$q=\hat{q}⊗q_{err}.$$

Assuming that the error quaternion, $q_{err}$, is to represent a small rotation, it can be approximated as follows:

(23) $$c={\left[q_{r}\;q_{x}^{\left(err\right)}\;q_{y}^{\left(err\right)}\;q_{z}^{\left(err\right)}\right]}^{T}={\left[q_{r}\;{\vec{q}}_{err}\right]}^{T}.$$

Noting that the error quaternion $q_{err}$ is a perturbation of the rotation matrix, and the vector components of ${\vec{q}}_{err}$ are small, the perturbation of the rotation matrix R in Equation (21) can be written as:

(24) $$R\left(q_{err}\right)≅\left[\begin{array}{ccc} 1 & -2q_{r}q_{z}^{\left(err\right)} & 2q_{r}q_{y}^{\left(err\right)}\\ 2q_{r}q_{z}^{\left(err\right)} & 1 & -2q_{r}q_{x}^{\left(err\right)}\\ -2q_{r}q_{y}^{\left(err\right)} & 2q_{r}q_{x}^{\left(err\right)} & 1 \end{array}\right]=I_{3x3}+2q_{r}{\left[{\vec{q}}_{err}\right]}^{x}.$$

Equation (22) relating $\hat{q}$ and ***q*** can be written as

(25) $$R\left(q\right)=R\left(\hat{q}\right)R\left(q_{err}\right)=R\left(\hat{q}\right)\left[I_{3x3}+2q_{r}{\left[{\vec{q}}_{err}\right]}^{x}\right].$$

$R\left(\hat{q}\right)$ is the estimate of the rotation matrix or the equivalent of $\hat{q}$. Now, considering the sensor frame $u_{s}$ and the earth frame $u_{e}$, we have

(26) $$u_{e}=R\left(\hat{q}\right)\left[I_{3x3}+2q_{r}{\left[{\vec{q}}_{err}\right]}^{x}\right]u_{s}={\hat{u}}_{e}+2q_{r}{\left[{\vec{q}}_{err}\right]}^{x}u_{s}.$$

Thus, the measurement relation for the quaternion error is obtained:

(27) $$\Delta u_{e}≜u_{e}-{\hat{u}}_{e}=2q_{r}{\left[{\vec{q}}_{err}\right]}^{x}u_{s}.$$

Accordingly, given the error quaternion $q_{err}$ and the sensor frame $u_{s}$, the perturbation of the earth frame $u_{e}$ can be described. The quantitative analysis of the error quaternion is detailed in Section 6.1.

### 4.2 Error Analysis

The analysis in Section 4.1 focuses on the quaternion error. Here we further explore the sensing measurement errors, which consist of the elements of the error quaternion.

Roll−Φ: rotation about the X-axisPitch−θ: rotation about the Y-axisHeading−ψ: rotation about the Z-axis

Now we associate a quaternion with Euler angles, which yields

(28) $$\hat{q}=\left[\begin{array}{c} -\sin\frac{\Phi }{2}\sin\frac{\theta }{2}\sin\frac{\psi }{2}+\cos\frac{\Phi }{2}\cos\frac{\theta }{2}\cos\frac{\psi }{2}\\ \begin{array}{c} +\sin\frac{\Phi }{2}\cos\frac{\theta }{2}\cos\frac{\psi }{2}+\cos\frac{\Phi }{2}\sin\frac{\theta }{2}\sin\frac{\psi }{2}\\ -\sin\frac{\Phi }{2}\cos\frac{\theta }{2}\sin\frac{\psi }{2}+\cos\frac{\Phi }{2}\sin\frac{\theta }{2}\cos\frac{\psi }{2} \end{array}\\ +\sin\frac{\Phi }{2}\sin\frac{\theta }{2}\cos\frac{\psi }{2}+\cos\frac{\Phi }{2}\cos\frac{\theta }{2}\sin\frac{\psi }{2} \end{array}\right]$$

Denote the pitch angle measurement as θ+Δθ, where θ is the true pitch angle information and Δθ is the measurement error. To simplify the error analysis, assume the rotation errors are neglected in roll angle and heading angle measurements. Let sin(Φ/2)=A and sin(ψ/2)=B. Accordingly, considering the measurement error in the pitch angle, the quaternion can be rewritten as

(29) $$q^{\prime }=\left[\begin{array}{c} -AB\sin\frac{\theta +\Delta \theta }{2}+\sqrt{\left(1-A^{2}\right)\left(1-B^{2}\right)}\cos\frac{\theta +\Delta \theta }{2}\\ +A\sqrt{1-B^{2}}\cos\frac{\theta +\Delta \theta }{2}+B\sqrt{1-A^{2}}\sin\frac{\theta +\Delta \theta }{2}\\ -AB\cos\frac{\theta +\Delta \theta }{2}+\sqrt{\left(1-A^{2}\right)\left(1-B^{2}\right)}\sin\frac{\theta +\Delta \theta }{2}\\ +A\sqrt{1-B^{2}}\sin\frac{\theta +\Delta \theta }{2}+B\sqrt{1-A^{2}}\cos\frac{\theta +\Delta \theta }{2} \end{array}\right]$$

Assuming that the measurement error in the pitch angle is small, we obtain

(30) $$\begin{array}{ccc} \sin\frac{\theta +\Delta \theta }{2} & = & \sin\frac{\theta }{2}\cos\frac{\Delta \theta }{2}+\cos\frac{\theta }{2}\sin\frac{\Delta \theta }{2}\\ & ≃ & \sin\frac{\theta }{2}+\cos\frac{\theta }{2}\cdot \frac{\Delta \theta }{2} \end{array}$$

(31) $$\begin{array}{ccc} \cos\frac{\theta +\Delta \theta }{2} & = & \cos\frac{\theta }{2}\cos\frac{\Delta \theta }{2}-\sin\frac{\theta }{2}\sin\frac{\Delta \theta }{2}\\ & ≃ & \cos\frac{\theta }{2}-\sin\frac{\theta }{2}\cdot \frac{\Delta \theta }{2}. \end{array}$$

According to Equation (29), the quaternion with measurement error in the pitch angle can be further approximated by

(32) $$\begin{array}{ccc} q^{\prime } & ≃ & \left[\begin{array}{c} -AB\sin\frac{\theta }{2}+\sqrt{\left(1-A^{2}\right)\left(1-B^{2}\right)}\cos\frac{\theta }{2}-AB\cos\frac{\theta }{2}\cdot \frac{\Delta \theta }{2}-\sqrt{\left(1-A^{2}\right)\left(1-B^{2}\right)}\sin\frac{\theta }{2}\cdot \frac{\Delta \theta }{2}\\ \;+A\sqrt{1-B^{2}}\cos\frac{\theta }{2}+B\sqrt{1-A^{2}}\sin\frac{\theta }{2}-A\sqrt{1-B^{2}}\sin\frac{\theta }{2}\cdot \frac{\Delta \theta }{2}+B\sqrt{1-A^{2}}\cos\frac{\theta }{2}\cdot \frac{\Delta \theta }{2}\\ -AB\cos\frac{\theta }{2}+\sqrt{\left(1-A^{2}\right)\left(1-B^{2}\right)}\sin\frac{\theta }{2}+AB\sin\frac{\theta }{2}\cdot \frac{\Delta \theta }{2}+\sqrt{\left(1-A^{2}\right)\left(1-B^{2}\right)}\cos\frac{\theta }{2}\cdot \frac{\Delta \theta }{2}\\ \;+A\sqrt{1-B^{2}}\sin\frac{\theta }{2}+B\sqrt{1-A^{2}}\cos\frac{\theta }{2}+A\sqrt{1-B^{2}}\cos\frac{\theta }{2}\cdot \frac{\Delta \theta }{2}-B\sqrt{1-A^{2}}\sin\frac{\theta }{2}\cdot \frac{\Delta \theta }{2} \end{array}\right]\\ & = & \hat{q}⊗q_{err} \end{array}$$

Note that, given a measurement error in the pitch angle, and the roll, pitch, and heading angle measurements, the error quaternion $q_{err}$ can be approximately derived by Equation (32). Therefore, the measurement relation with the quaternion error between the earth frame and the sensor frame can be further described using Equation (27). 

## 5. System Operating Procedures

To evaluate the effectiveness and capability of the virtual reality simulator for team training, we designed a between-subjects study. In the experiments, the impacts of three key factors on system performance are considered: training experience, the group size of the participants, and information exchange between the group members. The experimental results are detailed as follows.

### 5.1. Participants

The experiment involved 6 participants. No participants had ever played the system before. Half of the participants were volunteers who had done military training in live situations, while the other half had never done live training. The age of the participants ranged from 27 to 35.

### 5.2. Virtual Environment

**Figure 15:**
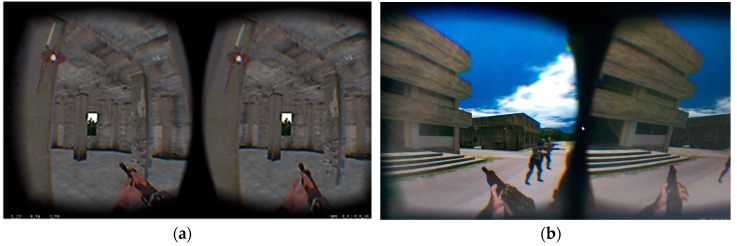
Virtual environment on a HMD. (**a**) An indoor view. (**b**) An outdoor view.

The immersive environment was designed for a rescue mission in an enemy-held building (Figure 15). In addition, three hostages were being guarded on the top floor of a three-storied building which was controlled by 15 enemies. To ensure that the virtual environment was consistent with an actual training facility, we simulated a real military training site, including the urban street, the enemy-held building, and so on. All enemies controlled automatically by the system were capable of shooting, evading attacks, and team striking. When participants were immersed in the virtual environment, they could interact with other participants not only through gesture tracking, but also through VoIP communication technology.

**Figure 16:**
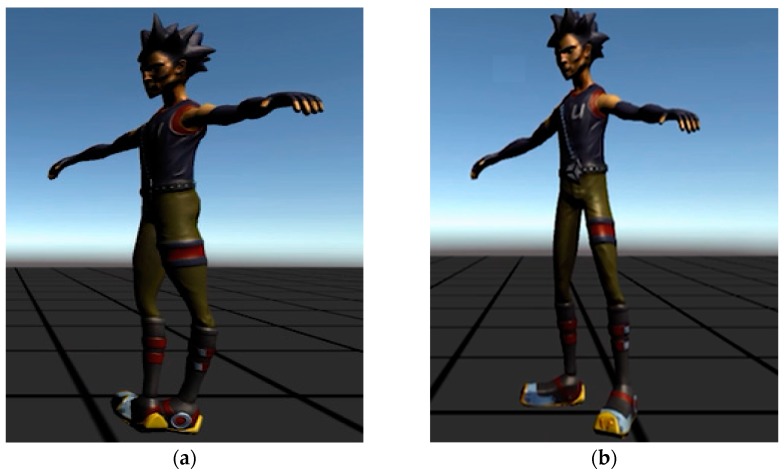
Sensing measurement errors of the sensor nodes were calibrated when the T-pose calibration was performed. (**a**) All sensor nodes were calibrated well during the T-pose procedure. (**b**) One sensor node attached to the right thigh was not calibrated well, and a sensing error was derived in the pitch direction.

As mentioned above, sensing measurement errors greatly affect the sensor nodes, which are attached to a human body. The integration of the inertial sensors, including sensor signals and drift errors, is performed on the basis of the kinematics of the human body. Therefore, sensing errors will be accumulated in quaternion form. As shown in Figure 16, the sensing measurement errors are calibrated when the T-pose calibration is performed. In the first step, we normalize the accelerometer, gyroscope and magnetometer in all of the sensor nodes and compensate for bias errors. In the following step, a complementary filter is used to mitigate the accumulated errors based on the advantages of long-term observation and short-term observation respectively. In the final step, T-pose calibration is performed to align the orientation of the sensor nodes with respect to the body segments, after which the sensor node is able to capture body movement accurately in the virtual environment.

### 5.3. Procedure

The purpose of the experiment is to evaluate the system performance. Participants follow the same path through the virtual environment in the experiment. The time-trial mission starts when the participants begin from the storm gate. Moreover, the time taken by the participants to kill all of the enemies who guard the three hostages on the top floor will be recorded. All participants control simulated M4 rifles, and the enemies control virtual AK47 rifles. All of the weapons have unlimited ammo. Under these experimental conditions, the participants’ death rate (hit rate) is recorded for data analysis by the experimenters. Moreover, if all participants are killed before they complete the mission, the rescue task is terminated, and the time will not be recorded for the experiment.

## 6. Experimental Results

In order to assess the system performance, four sets of experiments were performed to explore the impact of quaternion error and the training experience on mission execution and management. 

### 6.1. Error Analysis

**Figure 17:**
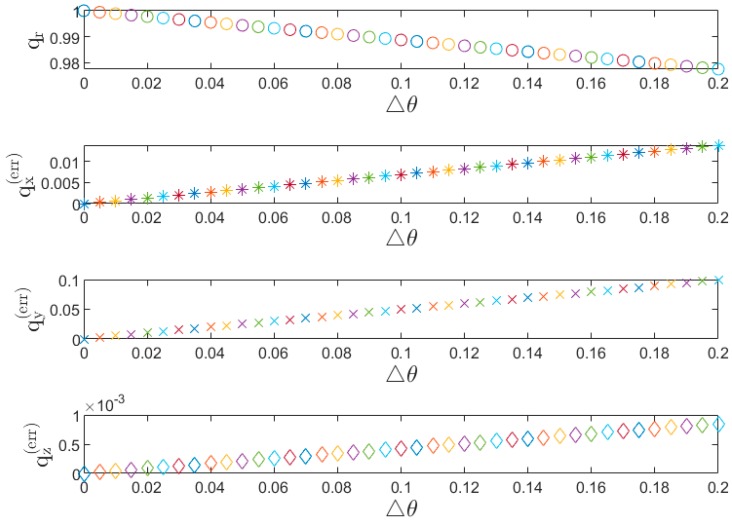
The scalar part and the vector part of the quaternion error for a small rotation of measurement error of pitch angle.

In the first set of simulations, we explored the characteristic of the error quaternion $q_{err}$. With reference to the analysis in Section 4.2, the rotation errors are assumed to be negligible in the roll angle and heading angle measurements, and the measurement error in the pitch angle is considered to be Δθ. With angle information (e.g., the heading angle $60^{{}^{\circ}}$, the roll angle $30^{{}^{\circ}}$, the pitch angle $15^{{}^{\circ}}$) and Δθ, Figure 17 presents the behavior of the error quaternion $q_{err}$ when varying the measurement error of the pitch angle. Note that, given $\Delta \theta \;\mathrm{ranging}\;\mathrm{from}\;0^{{}^{\circ}}\;\mathrm{to}\;0.2^{{}^{\circ}}$, the vector parts of $q_{err}$ (i.e., $q_{x}^{\left(err\right)},\;q_{y}^{\left(err\right)},\;q_{z}^{\left(err\right)}$) are approximately linear with respect to the Δθ, which can provide a sensible way of describing the error behaviors of rotation X, rotation Y, and rotation Z. According to Equation (27), given the quaternion error $q_{\mathrm{err}}$ and the sensor frame $u_{s}$, the perturbation of the earth frame $u_{e}$ can be described. As shown in Figure 17, when the measurement error in the pitch angle is small, the small vector components of ${q\;\to }_{\mathrm{err}}$ lead to a small perturbation of the earth frame $u_{e}.$ In contrast, as the measurement error in the pitch angle increases, the perturbation in the Y-axis increases, which results in a larger error component in the Y-axis (e.g., with Δθ=0.1, $q_{x}^{\left(err\right)}=0.006,\;q_{y}^{\left(err\right)}=\;0.05,\;q_{z}^{\left(err\right)}$ = 0.0004).

### 6.2. Simulated Training Performance

**Figure 18:**
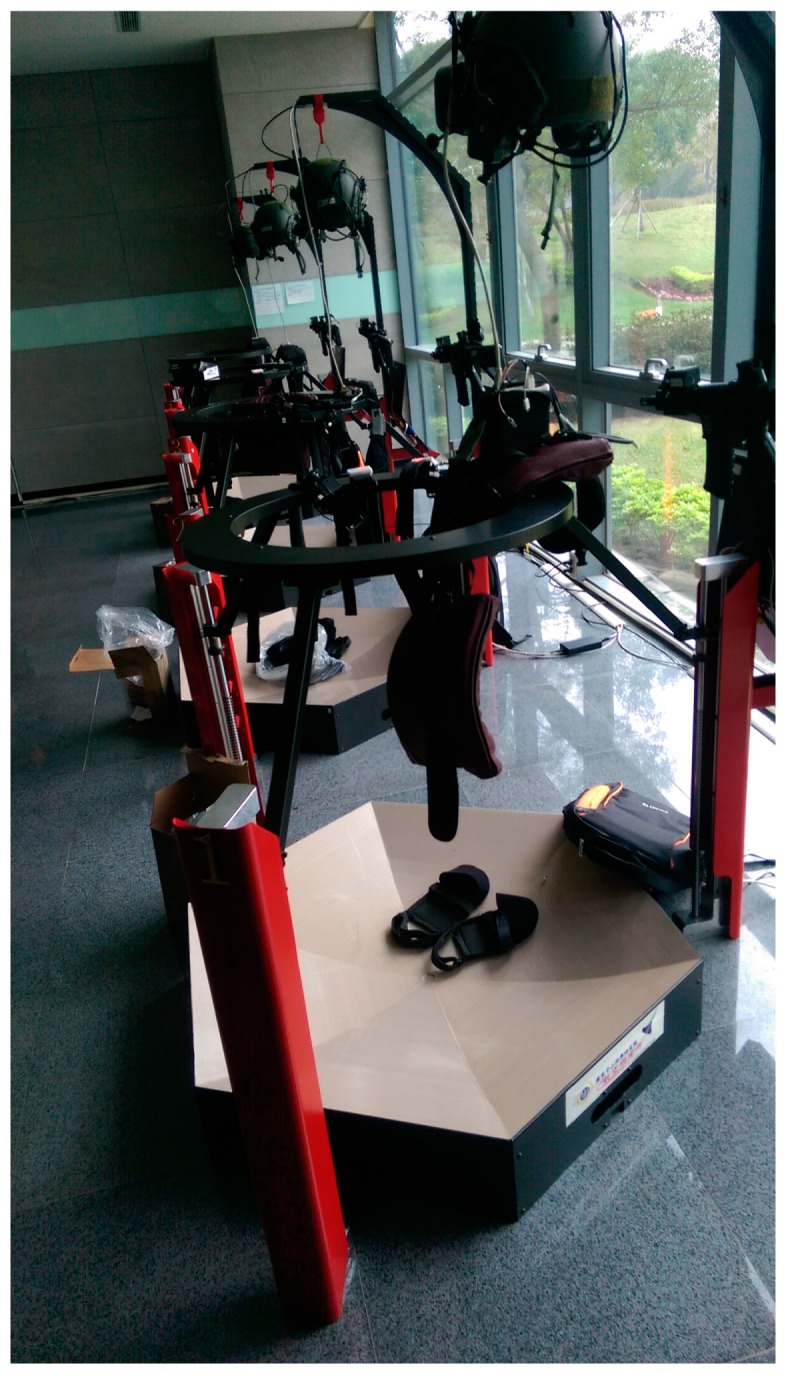
Snapshot of the system.

**Figure 19:**
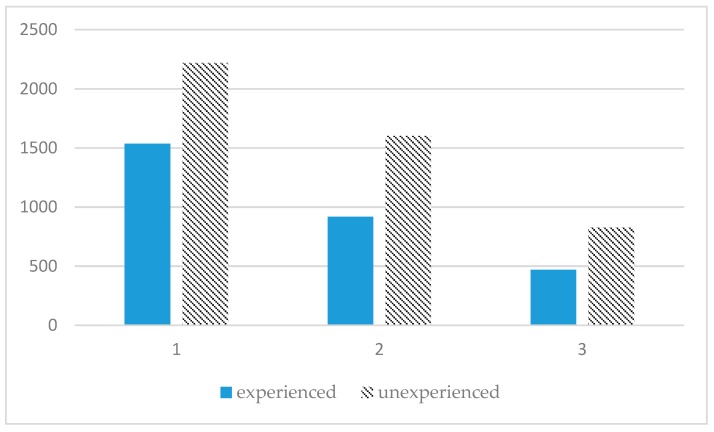
Means of experienced participants and unexperienced participants under various experimental conditions (horizontal axis: single-man, two-man, three-man; vertical axis unit: seconds).

**Figure 20:**
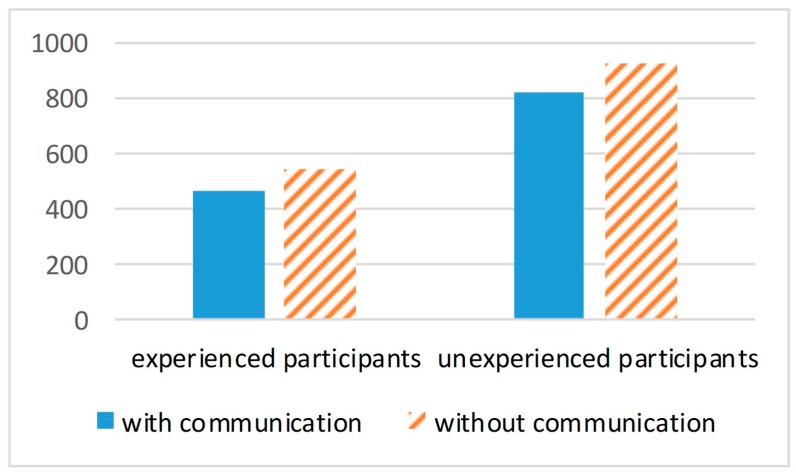
Mean of three-man teams with voice communication/without voice communication under the experimental conditions (horizontal axis: experienced/unexperienced participants; vertical axis unit: seconds).

Figure 18 shows a snapshot of the proposed system. All three individual participants, who had done the same military training at the actual training site, successfully completed the rescue mission, with times of 22′16″, 25′40″, 28′51″, respectively (mean = 25′36″, standard deviation = 3′18″). However, of the three participants who had never done the same training and started the mission individually, only one participant completed the rescue mission, with a time of 36′59″, and the other two participants were killed by enemies before completing the rescue task. The three two-man teams who had been trained in the live situation completed the rescue mission with times of 11′28″, 18′19″, 16′5″, respectively (mean = 15′17″, standard deviation = 3′30″). However, the three two-man teams who had never done the same training also completed the rescue mission, with times of 25′19″, 28′33″, 26′12″, respectively (mean = 26′41″, standard deviation = 1′40″). Finally, the three-man team that had live training experience completed rescue mission with a time of 7′49″. On the other hand, the three-man team that had never done the same training completed the rescue mission with a time of 13′47″. The results of experienced and unexperienced participants’ mean times in the experiment are shown in Figure 19. We also evaluated another situation, in which two subjects in the three-man groups completed the mission without the VoIP communication function. The mean time in this experiment increased by 1′26″ (Figure 20), which implies that communication and information processing can improve the performance for rescue missions.

**Figure 21:**
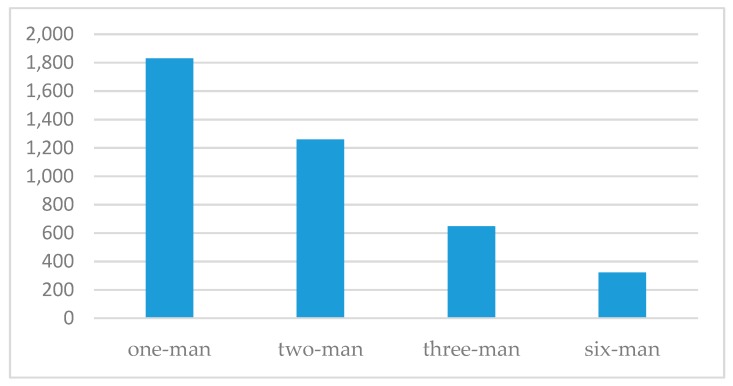
Mean times with different numbers of participants under the experimental conditions (horizontal axis: number of participants; vertical axis unit: seconds).

Finally, we evaluated a six-man team of all participants in the rescue task, because standard deployment for a real live mission is a six-man entry team. The mission time decreased by 2′28″ with respect to the three-man experiment with experienced participants. The experimental results for mean times with different numbers of participants are shown in Figure 21. In addition, death rate (hit rate) revealed another difference between single and multiple participants. From the results, the mean of death rate (hit rate) was 1.5 shots/per mission when a single participant interacted with the system. However, the mean of death rate decreased to 0.38 shots/per mission when multiple participants interacted with the system.

### 6.3. Discussion

When the participants executed the rescue mission, the activities involved in the experiment included detecting enemies, engaging enemies, moving inside the building and rescuing the hostages. The results reveal significant differences in several respects, including experience, quantity, and communication, and show that compared with the inexperienced participants, all experienced participants who had done the same training in a live situation took less time to complete the rescue mission. The wireless BANs of the participants are able to work accurately in the virtual environment for experienced participants. Tactical skills (e.g., moving through built-up areas, reconnoiter area, reacting to contact, assaulting, and clearing a room) absolutely require team work, demanding that wireless BANs interact with each other perfectly in terms of connection and accuracy. Without proper BANs, participants may feel mismatched interaction with their virtual avatars, and may feel uncomfortable or sick in the virtual environment.

The experimental results show that a larger sized group of participants took less time to complete the rescue mission than a smaller sized group of participants. Moreover, a group of multiple participants had a lower death rate compared with that of a single participant. This is due to the fact that, as the group size of participants increases, team movement is more efficient and fire power is greater in the virtual environment, which is similar to a real world mission. Furthermore, when the VoIP communication function was disabled, whether participants were experienced or not, the rescue mission time in the experiment consequently increased. As we know, in the real rescue mission, team coordination is important in the battlefield. In the system, all participants are able to interact with each other through hand signal tracking and voice communication. As a result, multiple-user training may become a key feature of the system.

## 7. Conclusions

In this paper, we have addressed problems arising when building an infantry training simulator for multiple players. The immersive approach we proposed is an appropriate solution that is able to train soldiers in several VR scenarios. The proposed simulator is capable of training six men at a system update rate of 60 Hz (i.e., the refresh time of the entire system takes about 16 ms), which is acceptable for human awareness with delay (≤ 40 ms). Compared with the expensive Xsens MVN system, the proposed simulator has a competitive advantage in terms of system cost. For future work, we intend to develop improved algorithms to deal with accumulated sensing errors and environment noise on wireless BANs. Consequently, the system can develop finer gestures for military squad actions and enrich the scenario simulation for different usages in military training. The system is expected to be applied in different kind of fields and situations.

## Figures and Tables

**Table 1:** The calibrated results of the sensor nodes.

	$CR_{acc}$	$CR_{mag}$	$CR_{gyro}$
**x**	0.95	0.98	0.96
**y**	0.97	0.99	0.96
**z**	0.99	0.98	0.94

**Table 2:** Data structure of a packet.

Header	Data to Show Packet Number	8 bits
**Payload**	Bone data size	144 bits
	Total data length	
	Soldier No.	
	T-Pose status	
	Sensor nodeID	
	Total Bones of a skeleton	
	Yaw value of the bone	
	Pitch value of the bone	
	Roll value of the bone	
**Tail**	Data to show end of packet	8 bits

**Table 3:** Description of the features of each soldier.

DATA	UNIT	RANGE
Soildier no.	N/A	0~255
Friend or Foe	N/A	0~2
exterbal Control	N/A	0/1
Team no.	N/A	0~255
Rank	N/A	0~15
Appearance	N/A	0~255
BMI	Kg/Meter^2^	18~32
Health	Percentage	0~100
Weapon	N/A	0~28
Vechicle	N/A	0~5
Vechicle Seat	N/A	0~5
Position X	Meter	N/A
Position Y	Meter	N/A
Position Z	Meter	N/A
Heading	Degree	−180~180
Movement	N/A	0~255
Behavior	N/A	0~255
